# Reasons for (Not) Seeking Care for Fatigue and Care Needs Among Patients With Inflammatory Bowel Disease: A Qualitative Interview Study

**DOI:** 10.1111/jan.16837

**Published:** 2025-02-25

**Authors:** Quirine M. Bredero, Milou M. Ter Avest, Gerard Dijkstra, Joke Fleer, Maya J. Schroevers

**Affiliations:** ^1^ Department of Health Sciences, Unit Health Psychology, University Medical Centre Groningen University of Groningen Groningen the Netherlands; ^2^ Centre for Mindfulness, Department of Psychiatry Radboud University Medical Centre Nijmegen the Netherlands; ^3^ Donders Institute for Brain, Cognition and Behaviour Radboud University Nijmegen the Netherlands; ^4^ Department of Gastroenterology and Hepatology Jeroen Bosch Hospital 's‐Hertogenbosch the Netherlands; ^5^ Department of Gastroenterology and Hepatology, University Medical Centre Groningen University of Groningen Groningen the Netherlands; ^6^ University College Groningen University of Groningen Groningen the Netherlands

**Keywords:** barriers and facilitators, care needs, fatigue, inflammatory bowel disease, nurse, qualitative research

## Abstract

**Aims:**

To explore reasons why patients with inflammatory bowel disease (IBD) do (not) engage in fatigue‐related care and their care needs.

**Design:**

A qualitative interview study, using a phenomenological methodological approach.

**Methods:**

We included 16 fatigued patients with IBD in remission. Data were collected between December 2021 and March 2022, using semi‐structured interviews. Template analysis was performed.

**Results:**

We identified six themes regarding reasons why (not) to seek care for fatigue: (1) Cognitions about fatigue and coping with fatigue, (2) perceptions of fatigue‐related care and previous care experiences, (3) perceived knowledge and behaviour of healthcare professionals, (4) physical and emotional well‐being, (5) social relationships and support, and (6) practical factors. Regarding their care needs, patients reported a need for a holistic and person‐centred care approach, with healthcare professionals actively addressing fatigue and offering care. They suggested a range of options for what care to offer, including eliminating physical causes of fatigue, discussing medication options, providing information on fatigue management, lifestyle support, psychological support, peer support and practical support.

**Conclusion:**

Both patient‐ and healthcare‐related factors play a role in why IBD patients do (not) seek fatigue‐related care. Our findings emphasise the importance of active screening and discussion of fatigue, using a holistic and person‐centred approach to treat fatigue.

**Implications:**

This study contributes to the understanding of IBD patients' facilitators and barriers for seeking care for fatigue and their care needs. Moreover, results can inform nurses and physicians about ways to optimise the offer and uptake of fatigue‐related care, and the development of interventions that fit patients' needs. Results also provide implications for the treatment of fatigue in other chronic (inflammatory) conditions.

**Impact:**

The current results can inform nurses and physicians about ways to optimise the offer and uptake of fatigue‐related care, and the development of interventions that fit the needs of patients with IBD. An increase in the uptake of effective and acceptable interventions can improve patients' health and well‐being.

**Reporting Method:**

Findings were reported following the consolidated criteria for reporting qualitative research (COREQ).

**Patient or Public Contribution:**

No patients, service users or members of the public were involved in this study. The study focused on patients' experiences with fatigue‐related care and their needs.

**Summary**

- Fatigue is one of the most prevalent and burdensome symptoms in patients with inflammatory bowel disease (IBD). Despite the availability of effective interventions for fatigue, the uptake of care is low.
- Patients with IBD report various personal‐ and healthcare‐related facilitators and barriers for seeking fatigue‐related care. Findings emphasise the importance of healthcare professionals actively addressing fatigue, using a holistic and person‐centred approach to treat fatigue.
- This study provides recommendations for nurses and physicians on how to address fatigue and improve fatigue‐related care, both for IBD and other chronic (inflammatory) conditions in which fatigue is a problem.

## 1 Introduction

Inflammatory bowel disease (IBD) includes Crohn's disease (CD) and ulcerative colitis (UC), two lifelong inflammatory conditions of the gastrointestinal tract. IBD is typically diagnosed between the ages of 20 and 40, and often follows an unpredictable relapsing–remitting course (Seyedian et al. 2019). Treatment aims to maintain remission and prevent complications; however, there is currently no cure for IBD. Fatigue is one of the most prevalent and persistent symptoms among patients with IBD (McGing et al. 2021). IBD‐related fatigue is often described as a persistent feeling of tiredness, accompanied by periods of sudden, intense exhaustion that cannot not be alleviated by rest or sleep (Czuber‐Dochan, Norton, Bassett, et al. 2014). The aetiology of fatigue in patients with IBD is assumed to be multifactorial, including active inflammation, nutritional deficiency, metabolomic alterations, lifestyle and psychological comorbidities (McGing et al. 2021; Borren et al. 2019). While managing IBD‐related fatigue typically involves alleviating disease activity (van Bodegraven et al. 2010), many patients continue to experience fatigue even during remission (D'Silva et al. 2021). The prevalence is around 47% of patients in remission and 72% with active IBD (D'Silva et al. 2021). Fatigue can have an extensive impact on patients' quality of life and psychosocial functioning (Radford et al. 2020), including diminished work functioning, reduced well‐being and symptoms of depression and anxiety (Czuber‐Dochan et al. 2013; Schreiner et al. 2021; Cohen et al. 2014). Due to its' complex nature, patients often spend a prolonged period searching for and developing effective self‐management strategies to cope with fatigue and related problems (Dibley et al. 2021).

Although effective interventions are available to reduce IBD‐related fatigue, including medication management and both psychological and physical therapies (Davis et al. 2020; Emerson et al. 2021), the uptake of care remains low (Bredero et al. 2023; Dhalaigh et al. 2021). A similar low uptake of care has been found in patients with IBD in the context of interventions for reducing psychological distress (Greywoode et al. 2023). These findings suggest that the experience of a symptom or problem such as fatigue does not necessarily translate into a need for, and uptake of, care. In order to address the low uptake of available effective interventions for IBD‐related fatigue, a deeper understanding is needed of patients' reasons for seeking or not seeking and accepting care (Byron et al. 2022). Moreover, since the low uptake of care might indicate that current care offers do not match the needs of patients with IBD, a better insight into their care needs is warranted. An increase in the uptake of effective and acceptable interventions can, after all, improve patients' health and well‐being.

## 2 Background

With the majority of studies focusing on the prevalence and predictors of fatigue, and the effects of interventions, knowledge on reasons for (not) seeking care for fatigue in patients with IBD is very limited. Regarding fatigue, studies have reported that many patients with IBD do not talk about it with their healthcare professional and feel dissatisfied with the attention and support received upon addressing it as an issue (Czuber‐Dochan et al. 2013; Beck et al. 2013; Dibley et al. 2020). So far, only one qualitative study in patients with IBD indicated that their need for care is related to their perceptions of fatigue (e.g., as an inevitable part of IBD or as something that cannot be treated) and perceived availability of care, as well as how they perceive healthcare professionals' attitudes and their satisfaction with the relationship with healthcare professionals (Dhalaigh et al. 2021). A few studies in patients with IBD did examine their need for interventions to reduce psychological distress and symptoms of depression and anxiety, reporting that important barriers to seeking this care were insufficient knowledge about available care services, limited access to mental healthcare, limited time, and costs of care uptake (Greywoode et al. 2023; Lawrence and Choudhary 2020).

Besides the current lack of knowledge on *why* patients with IBD do (not) seek care, little is known about their specific needs for care to reduce fatigue. The handful of studies that have examined this topic show that patients desire open, person‐centred communication about fatigue with physicians and nurses (Czuber‐Dochan et al. 2013; Beck et al. 2013; Dibley et al. 2020). This includes having their ideas, experiences and preferences valued and incorporated into a medical consultation and treatment decisions. At the same time, Beck and colleagues (2013) indicated that some patients do not feel the need to talk about fatigue unless the healthcare professional could treat the problem. These studies report valuable first insights into patients' need for addressing and discussing fatigue as an issue, however, no thorough investigation has yet been performed regarding their specific needs in terms of *what* type of support, and *how* it should be ideally provided. We expect that our results will yield a better understanding of ways to optimise the offer and uptake of fatigue‐related care for patients with IBD, guiding future researchers and IBD‐professionals in the development of intervention strategies that fit patients' needs.

## 3 This Study

### 3.1 Aims

This qualitative study aimed to explore: (1) patients' reasons for (not) seeking care for IBD‐related fatigue (i.e., facilitators and barriers) and (2) patients' care needs concerning IBD‐related fatigue.

## 4 Methodology

### 4.1 Design

This qualitative study was conducted based on an epistemological interpretivist/constructivist perspective, which assumes that reality is constructed through interactions between a researcher and the participant, and that researchers cannot conduct value‐free research by separating themselves from their previous experiences and interpretations (Snape and Spencer 2003). This perspective follows an ontological relativist paradigm, which accepts that there is no absolute truth and that reality is only knowable through socially constructed meanings (Snape and Spencer 2003). The methodological approach for the current study was phenomenological since we aimed to gain insight into the experiences and perceptions of patients regarding reasons for (not) seeking care and care needs, using in‐depth, semi‐structured interviews (Neubauer et al. 2019). The method of data analysis was template analysis (King and Brooks 2017). The reporting of this study conforms to COREQ guidelines (Tong et al. 2007) (Supporting Information 1).

### 4.2 Participants

We included patients diagnosed with either Crohn's disease (CD), ulcerative colitis (UC) or IBD‐unclassified (IBD‐U), who were aged between 18 and 75 years old. Patients were severely fatigued, as defined by a score ≥ 6 on the question “To what extent do you feel fatigued?” (scale 1–10) in their medical records and a score ≥ 35 on the subscale ‘subjective fatigue’ of the self‐report questionnaire Checklist Individual Strength (CIS‐8) (Vercoulen et al. 1994). Since we assumed that a disease flare could affect patients' current care need (e.g., a stronger need for medical treatment), we only included patients who were in remission. For UC and IBD‐U, remission was determined by a score ≤ 3.54 on the Monitor IBD At Home (MIAH) (de Jong et al. 2019) and < 2.5 on the Simple Clinical Colitis Activity Index (Walmsley et al. 1998). For CD, remission was determined by a score ≤ 3.62 on the MIAH (de Jong et al. 2019) and a score < 4 on the Harvey Bradshaw Index (Harvey and Bradshaw 1980). No exclusion criteria were used.

### 4.3 Data Collection

Data collection took place at the outpatient clinics of the department of Gastroenterology at the University Medical Centre Groningen (UMCG) between December 2021 and March 2022. All adult patients who were diagnosed with IBD and showed, based on their medical records, disease activity in remission and elevated levels of fatigue received an email with information about the study and an online screening questionnaire on fatigue, including the CIS‐8 (Vercoulen et al. 1994) and two questions asking whether they receive(d) care for fatigue and whether they have a need for professional care for fatigue. Sociodemographic variables (i.e., age, gender) and clinical information (i.e., type of IBD, date of diagnosis, medication use, and co‐morbidities) were collected to describe the sample.

**FIGURE 1:**
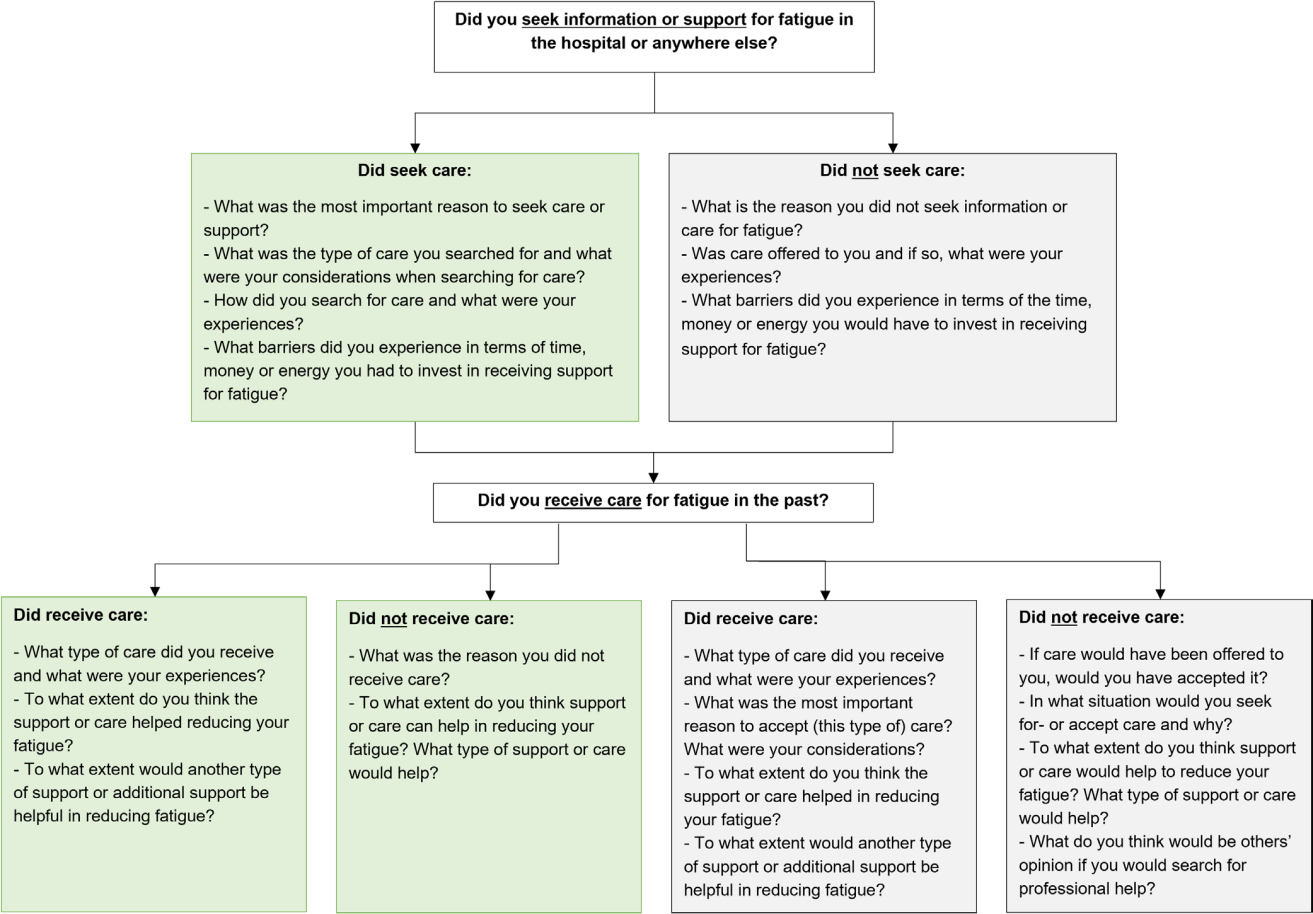
Interview topic guide.

All eligible patients were invited for the interview by email. The interviews were held via video calling (using Teams) due to COVID‐19 restrictions. They lasted between 55 and 70 min, with an average duration of 65 min. A semi‐structured interview schedule was followed (Figure 1), of which questions and probes were created by the first author (Q.M.B.), in collaboration with all co‐authors. The questions were based on previous qualitative studies examining care experiences and needs in patients with IBD (Czuber‐Dochan et al. 2013; Sweeney et al. 2019) and on clinical experience. The interview guide was used in all interviews, and the interviewer was free to ask follow‐up questions or divert from the structure of the interview guide where she deemed this necessary or appropriate. As specified in the topic guide, different questions were selected, depending on whether patients sought and/or received care. No repeat interviews were carried out.

Before starting the data collection, two pilot interviews were performed in order to practice the interviews with patients with IBD, review the interview schedule, and assess data quality (i.e., richness of data and fit with research question). All interviews were performed by Q.M.B., a female PhD candidate and psychologist trained in conducting interviews and leading a research project on IBD‐related fatigue. The interviewer had no relationship with the participants prior to the study, and no other non‐participants were present. The interviewer had access to the results of the screening questionnaire completed by the patients in order to ask more direct questions about aspects of the disease, fatigue, and the context relevant to the patient (e.g., their diagnosis). Field notes were taken during the interviews. All interviews were recorded via Teams and anonymised and transcribed verbatim by Q.M.B. and three research assistants.

After performing 16 interviews, we stopped recruiting patients. Considering the combination of a strong and focused dialogue providing a large amount of in‐depth data, a narrow study aim, and the focus on a specific patient group, we decided that these interviews provided sufficient information power (Malterud et al. 2015). Generally, the concept of information power indicates that the more information the sample holds, the lower the number of participants is needed.

### 4.4 Data Analysis

We performed template analysis (King and Brooks 2017), a method that features a structured coding process, early theme development, and the conceptualization of themes as topic summaries. We chose this approach due to our focused research questions on reasons (not) to seek care and care needs. Moreover, a codebook approach is practical when multiple coders work on different parts of the data. ATLAS.ti (version 24) was used to organise and store the data. The analysis orientation was both inductive (codes were developed based on the content of data) and deductive (a codebook was used early on in the analysis process). We closely followed the seven steps as developed by King and Brooks (2017). First, Q.M.B. read through all transcripts and listened to the audio recordings to familiarise with the data. Similarly, M.J.S. (a female associate professor in health psychology), M.M.T.A. (a female PhD candidate and physician) and three research assistants who assisted in the coding process familiarised with (a subset of) the data. Coding was performed by multiple coders to enhance reflexivity and interpretative depth. Second, Q.M.B. and M.J.S. coded two interviews by highlighting points of interest relevant to the research questions and discussed and agreed on potential codes. Third, based on these codes, four a priori themes were developed to cluster the codes: “Reasons to seek care,” “Reasons to not seek care,” “Care needs” and “Care experiences.” Fourth, an initial coding template was developed, including these four themes in a non‐hierarchical manner. Fifth, the initial template was then applied to all other interviews by Q.M.B. and M.M.T.A. together with three research assistants. During this process, new codes were added and codes were aggregated or deleted if agreed upon. After applying the initial template to all interviews, Q.M.B. and M.M.T.A. added subthemes to the template and changed the structure of themes into the structure as reported in the results section. The final template (Supporting Information 2) was discussed with all co‐authors. Sixth, when all data relevant to the research questions were covered in the final template, we applied the template to the full dataset by going over all interviews and checking the coding once again. Finally, this template was used to develop an interpretation of the data and present it in the current paper.

### 4.5 Ethical Considerations

The study was approved by the Institutional Review Board of the University Medical Centre Groningen (UMCG) on July 12, 2021, registered as no. 2021/329, and the study has been performed in accordance with the principles of the Declaration of Helsinki (2013). The invitation to the study was sent via email and included an information letter explaining that participation was voluntary. All patients provided written informed consent for their participation in the study.

### 4.6 Rigour and Reflexivity

To establish rigour in this study, we used four criteria: credibility, transferability, dependability and confirmability (Ahmed 2024). Credibility was ensured through the researcher's background as a PhD candidate specialising in IBD‐related fatigue, resulting in extensive knowledge of the disease and study population. Additionally, the semi‐structured interview format enabled flexibility, allowing the interviewer to deepen her understanding of participants' responses as needed. Finally, credibility was established through reflexivity, which involved discussing personal biases and preconceptions within the research team. Transferability was addressed through clear inclusion criteria and purposive sampling, resulting in a diverse group of patients varying in age, gender, and type of disease, though with a slight predominance of females. Participants were recruited from an academic hospital, covering a broad geographical area in the Netherlands. Data collection took place online during the COVID‐19 pandemic, which may have influenced patients' participation in the study. Dependability was ensured by closely adhering to the template analysis method and documenting study decisions in memos. Lastly, confirmability was achieved through peer debriefing, where research findings were discussed, and feedback was sought from colleagues within and outside the research team.

## 5 Findings

### 5.1 Characteristics of Participants

In total, 135 patients with IBD received an email with information about the study and an online screening questionnaire on fatigue, of which 31 completed this questionnaire. Based on the screening, one patient was excluded because she did not complete the informed consent, and six were excluded because they were not severely fatigued (CIS‐8 < 35). Twenty‐four patients were invited for an interview, of which eight refused to participate or did not respond to the invitation.

**FIGURE 2:**
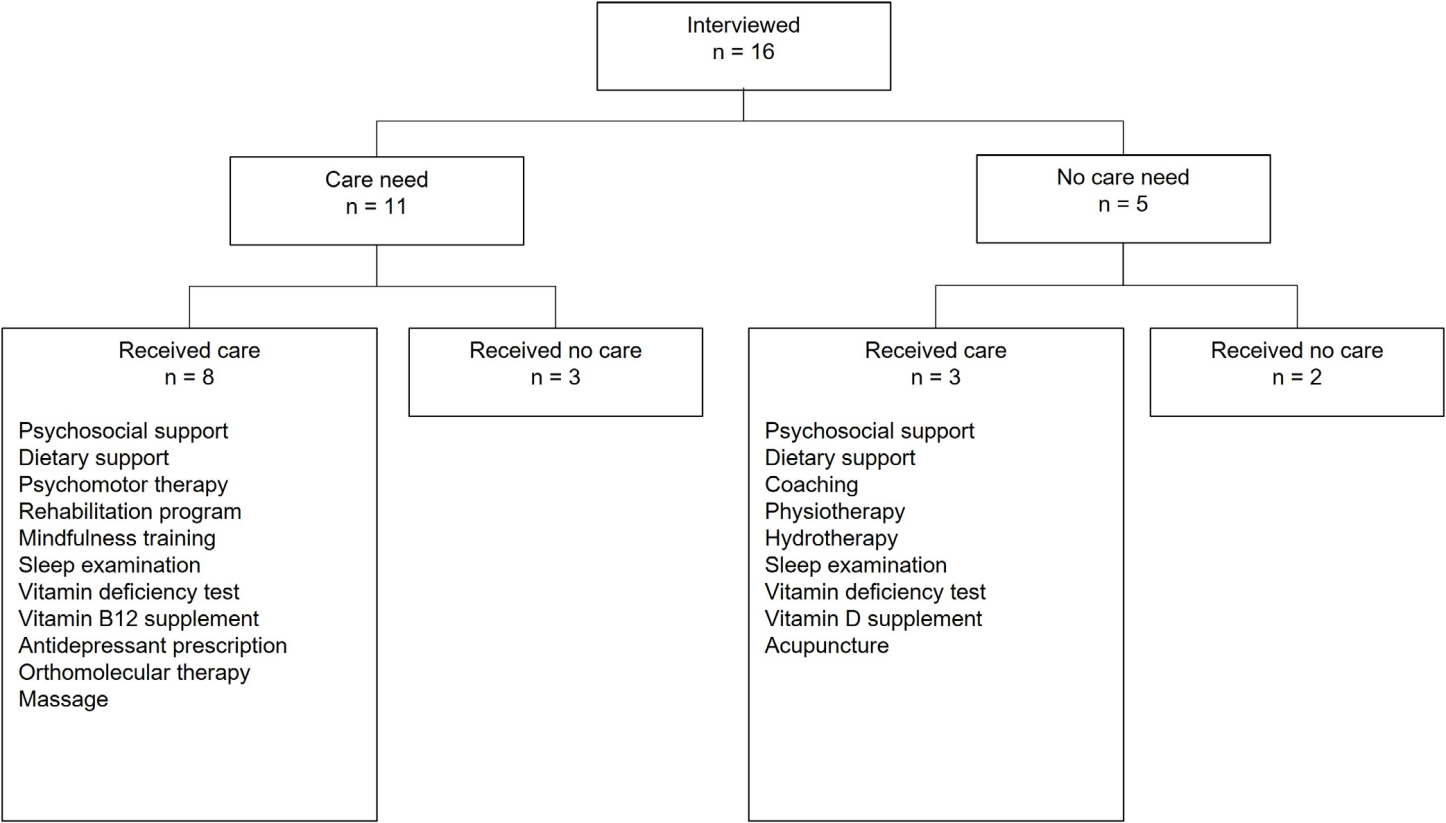
Overview of respondents' care needs and received care as reported during the interviews.

We interviewed 16 patients with IBD between 24 and 64 years old, including 12 females (Table 1). Nine patients were diagnosed with CD, six patients with UC, and one with IBD‐U. All patients used maintenance medication for IBD, and 13 reported a comorbid somatic disease such as diabetes mellitus, chronic obstructive pulmonary disease, fibromyalgia and skin diseases. Figure 2 shows an overview of patients' current need for care and received care.

**TABLE 1:** Respondent overview (*n* = 16).

Respondent*	Gender	Age (years)	Diagnosis	Time since diagnosis (years)
Rachel	Female	26	CD	12
Mary	Female	65	UC	33
Thomas	Male	33	UC	10
Linda	Female	55	CD	6
Sarah	Female	31	CD	7
Daniel	Male	24	CD	11
Laura	Female	52	UC	6
Karen	Female	54	UC	21
Melissa	Female	43	IBD‐U	14
Natalie	Female	43	CD	27
Hannah	Female	33	CD	15
Brian	Male	46	CD	25
Nicole	Female	35	UC	16
Sandra	Female	49	UC	6
Jennifer	Female	53	CD	23
Paul	Male	59	CD	26

Abbreviations: CD, Crohn's disease; CIS‐8, Checklist Individual Strength—8‐item subjective fatigue scale (score range 8–56, score ≥ 35 indicates severe fatigue); CU, ulcerative colitis; IBD‐U, IBD‐unclassified.

* Patients' fictional name.

### 5.2 Reasons for (Not) Seeking Care: Facilitators and Barriers

From the interviews, we identified six themes regarding reasons to (not) seek care for fatigue: (1) cognitions about fatigue and coping, (2) perceptions of fatigue‐related care and previous care experiences, (3) perceived knowledge and behaviour of healthcare professionals, (4) physical and emotional well‐being, (5) social relationships and support, and (6) practical factors. Within each of these themes, we classified the reasons into facilitators and barriers (Table 2). The facilitators and barriers are described below and illustrated with quotes from patients. A full overview of illustrative quotes can be found in Supporting Information 3.

**TABLE 2:** Facilitators and barriers for seeking care.

Themes	Facilitators	Barriers
Cognitions^a^ about fatigue and coping	*Fatigue* Perceiving fatigue as too hindering *Coping* Desiring to improve functioning Perceiving to be unable to deal with fatigue independently Seeking a medical explanation for fatigue	*Fatigue* n/a *Coping* Perceiving to have learned to live with fatigue Desiring to deal with fatigue independently Denying having a chronic disease
Perceptions of fatigue‐related care and previous care experiences	Having trust in the effectiveness of available care	Having limited trust in the effectiveness of available care Perceiving care needs are fulfilled Perceiving that nothing can be done about fatigue Having a lack of knowledge
Perceived knowledge and behaviour of healthcare professionals	Perceiving fatigue as a topic of conversation	Perceiving that fatigue is not taken seriously by healthcare professionals Perceiving that fatigue‐related care is not (adequately) offered Perceiving lack of knowledge in healthcare professionals regarding how to deal with fatigue
Physical and emotional well‐being	Experiencing (other) physical symptoms Experiencing an unhealthy lifestyle	Experiencing IBD‐related physical symptoms Feeling emotionally unfit
Social relationships and support	Perceiving negative impact of fatigue on relationships Receiving advise from others to seek care	n/a
Practical factors	n/a	Perceiving practical barriers

^a^ Cognitions refer to illness perceptions, cognitive emotion regulation and ways of coping.

#### 5.2.1 Theme 1: Cognitions About Fatigue and Coping

##### 5.2.1.1 Facilitators

Patients expressed that fatigue often negatively affects their ability to function at the level of peers in various areas of their lives, including work, taking care of their children, relationships, and mental well‐being. Especially when they *perceived that fatigue had become too hindering*, it prompted them to seek care. This notion was strongly related to *the desire to improve functioning*. As illustrated by the quote below, patients expressed their hope to return to work or school, and they reported a strong desire to regain a sense of “normalcy”, that is, to be able to perform tasks and activities they could engage in before the onset of their illness.

I want it to be as optimal as possible, so I always try to take that step to get back towards normal. Not so much in terms of illness, but just in terms of functioning, in daily activities and things you want to do. … You don't want the disease to take over in that. [Thomas]

Several patients expressed their intention to seek help as they *felt unable to deal with fatigue independently*:

Especially the consideration that I can't do that alone. I was thinking ‘I couldn't see the forest for the trees. What do I need to do to feel better?’ [Linda]

A final coping‐related facilitator for seeking care included *seeking a medical explanation for fatigue*. Patients hoped that identifying the cause would lead to effective treatment. They underwent various medical examinations to assess potential underlying factors of fatigue, including disease activity, vitamin deficiencies, allergies, or a sleep disorder.

I also had a test done via the general practicioner, just for sleep apnea, because they thought maybe the problem is quality of sleep. Nothing came out of it. [Daniel]

##### 5.2.1.2 Barriers

Several patients described that they *had learned to live with fatigue*, either because they accepted fatigue as part of their life or because it did not hinder them enough in daily life to discuss it with their healthcare professional.

It's always bothering me, it's not something I've had trouble with just recently. So I've kind of learned to live with it actually. In that sense, it doesn't hinder me so much that I would go and get help for that. [Rachel]

Some patients put the burden of fatigue into perspective by comparing it to more severe pain or a severe life event. Others explained that they adapted their lives to fatigue, for example, by quitting their jobs and limiting daily activities to a minimum. They described ongoing efforts to find a balance between pursuing what they find important while considering their low energy levels. A few patients experienced difficulties in managing fatigue, yet held the belief that they *needed to deal with fatigue independently*. They felt they needed to take care of themselves as much as possible before seeking additional support for fatigue. This idea was often based on the belief that others would criticise or ridicule them for not being able to deal with fatigue alone.

*Denying having a chronic disease* was considered another barrier for seeking care. Some patients refused to acknowledge their illness, ignoring signs of fatigue and pushing themselves beyond their boundaries. Others avoided addressing IBD‐related matters, simply because they were weary of medical issues and hospital visits.

I'd rather immerse myself in the things I enjoy, in hobbies and so on, than another medical problem, or another thing I don't really want to deal with. [Natalie]

#### 5.2.2 Theme 2: Perceptions of Fatigue‐Related Care and Previous Care Experiences

##### 5.2.2.1 Facilitators

Patients who had not received care reported that they would consider doing so if they *trusted the effectiveness of available care*. They primarily weighed the experienced burden of fatigue against the time and energy required to receive care and the expected effectiveness of the treatment. Up to this point, they perceived that engaging in care was not worth the investment.

If it's really something with a high chance of success, it would be different. But to go somewhere and they don't really know what to do with you, that's a little tricky. [Brian]

A few patients emphasised the importance of receiving information on the scientific evidence of a certain treatment, as this would aid them in making informed decisions.

##### 5.2.2.2 Barriers

In line with this, *having limited trust in the effectiveness of available care* for fatigue was a barrier to seeking care. Negative experiences with psychological treatment or peer support groups, for example, because they proved ineffective or demanded an excessive investment of energy and time, led to hesitation in pursuing a similar type of care. One patient reported that her trust in medical staff was limited due to past mistakes. Another patient reported currently having no need for care because his *care need was already fulfilled*.

Patients also mentioned that they did not seek care because they *believed that nothing could be done about fatigue*. This perception frequently stemmed from information communicated by healthcare professionals or other patients with IBD regarding fatigue and its related care, such as that fatigue is part of IBD. As one patient explained:

They have been saying all the time: ‘This fatigue, it's just part of it [IBD] and we don't really know what to do with it.’ So you just push that aside first, I guess. [Natalie]

Another barrier was *having a lack of knowledge* regarding fatigue‐related care. Several patients mentioned that they had not considered the option of seeking care for fatigue because they were unaware of available treatment options. Some interpreted a lack of information as an indication that there are no treatment options available, whereas others contemplated seeking help but were uncertain about what would be beneficial and whom to ask.

I never thought about it, because I wouldn't know who to turn to. There's not much information about it, in my opinion. I haven't seen or found that yet. [Sandra]

#### 5.2.3 Theme 3: Perceived Knowledge and Behaviour of Healthcare Professionals

##### 5.2.3.1 Facilitators

The opportunity to *discuss fatigue* during a medical consultation was perceived as a strong facilitator for seeking care. Specifically, patients reported that it was helpful when a healthcare professional addressed fatigue as a topic, offering insights into treatment options or scientific research related to interventions for IBD‐related fatigue.

From the moment I ended up in the [name of hospital], my request for help for fatigue came up a lot more. I was actually advised in that by the physician himself. Those questions were asked much more concretely here, compared to in my previous hospital. [Thomas]

##### 5.2.3.2 Barriers

Similarly, a perceived barrier to seek care was that *fatigue was not taken seriously* during consultations with physicians. Patients noted instances where healthcare professionals either failed to respond to fatigue complaints or disregarded fatigue as an untreatable problem.

Of course, during check‐ups they always ask how you feel. I always indicate that I'm tired, but they either don't respond or they say ‘Well, that's part of it.’ […] So I didn't really ask for it, no. [Rachel]

Moreover, even when physicians acknowledged fatigue, *care for fatigue was not (adequately) offered*. Some patients mentioned that care was not offered at all, whereas others received advice that did not align with their needs. For instance, suggestions regarding improving sleep with medication were offered, even though the patient feared potential dependency. Such experiences have contributed to the perception that *healthcare professionals also do not know how to deal with fatigue*, a perception occasionally validated by the healthcare professionals themselves.

Often I hear from them: ‘Yes okay, it's too bad you are fatigued, but we're not so sure what to do with that either…’ [Nicole]

#### 5.2.4 Theme 4: Physical and Emotional Well‐Being

##### 5.2.4.1 Facilitators

Two patients who received care in which the treatment of fatigue was integrated (i.e., psychological care and a rehabilitation program) explained that their initial reason for seeking care was *experiencing (other) physical symptoms*, such as weight loss and pain. They found that these treatments also had positive effects on their fatigue. Additionally, patients who associated *their unhealthy lifestyle* with fatigue, such as eating sugary foods and lacking physical activity, reported seeking support to adopt a healthier lifestyle as a reason for seeking care.

In the time I've been at home, I've gained a little bit of weight. So I also did seek help from dietitians, as I'm trying to lose some weight. Hopefully that also helps with getting a little more energy. [Daniel]

##### 5.2.4.2 Barriers

On the other hand, *experiencing physical symptoms of IBD* was reported as a barrier to seek care. For example, patients reported that abdominal pain and incontinence withheld them from leaving the house to receive support. Another perceived barrier to seek care was *feeling emotionally unfit*, as this made it difficult to take action upon a need for care.

So when things are back on track a little, there will be room for me to undertake things again. But if I'm not feeling well, physically or mentally, there's no room to take the initiative. [Melissa]

#### 5.2.5 Theme 5: Social Relationships and Support

##### 5.2.5.1 Facilitators

When being fatigued had a *negative effect on their partner and family relationships*, this was a facilitator for seeking help. One patient reported that he sought professional psychological care to free his partner from the burden of talking about his fatigue. Another patient felt that she was burdening her family members by functioning suboptimally in daily life.

I noticed that it was all taking too long and that I was burdening my family. As soon as I notice it's getting really annoying, also for everyone around me, I'm going to make an effort to get help. [Melissa]

A second social facilitator was when *seeking care was advised by others*, including advice from a family member or friend who had had good experiences with a certain type of care or knew about people who benefitted from care.

No barriers were reported within this theme.

#### 5.2.6 Theme 6: Practical Factors

##### 5.2.6.1 Barriers

*Practical barriers* for seeking care mostly included the required time and timing of care throughout the day. Moreover, distance and costs associated with receiving additional support for fatigue were also considered an issue.

That [care for fatigue] is mostly in the south of the country. There's actually too little here in the north. I am not going to [city in the south] just because we're all going for a walk there. I would love to, but no, that's too far for me. [Nicole]

### 5.3 Care Needs for IBD‐Related Fatigue

Based on patients' needs and suggestions on how to improve current practice for IBD‐related fatigue, we identified two main themes: (1) how to offer care, and (2) what care to offer, each including several subthemes (Table 3). Patients' care needs are described below and illustrated with quotes.

**TABLE 3:** Care needs for IBD‐related fatigue.

Themes	Subthemes
How to offer care	Taking a person‐centred approach Taking a holistic care approach Healthcare professionals should discuss fatigue and offer care actively
What care to offer	Information provision on fatigue management Eliminating physical causes for fatigue Discussing medication options Lifestyle support Psychological support Peer support Practical support

#### 5.3.1 Theme 1: How to Offer Care

Many patients stressed the importance of addressing their individual needs (i.e., *a person‐centred approach*) in the care for IBD‐related fatigue, especially regarding the type, timing of offering, and location of care. As illustrated by the quote below, patients often felt unsure how to find the type of care that may be helpful to them.

On the internet there is plenty to find. You are overloaded with all kinds of success stories and that just makes you sad. You don't know how to handle it. Where to start? … You just want what's right for you. [Natalie]

Patients also explained that some may require psychological support, whereas others benefit more from practical support such as household support or childcare. Some specifically preferred support outside the medical setting. It was also suggested to provide information on fatigue‐related care early after diagnosis, as patients then struggle to adapt to living with a chronic disease. Conversely, practical support, such as household support or financial support, was deemed more beneficial in later stages when fatigue persists. Regarding the location of care, patients found it important for care to be available close to home.

In addition, patients reported a need for a *holistic care approach*. They noted that physicians often concentrate primarily on treating flares or keeping the disease in remission, without considering their overall well‐being (including fatigue) and the psychological impact of a chronic illness. Additionally, patients were disappointed by the lack of communication between disciplines in the hospital. For example, those with comorbidities felt that healthcare professionals often overlook the interrelatedness of symptoms across diseases, whereas patients themselves often perceive fatigue as a common symptom of chronic diseases. Moreover, it was mentioned that psychologists lack sufficient medical knowledge to fully understand the burden of IBD. Patients suggested that facilitating communication between disciplines and taking a broader perspective on their symptoms could significantly enhance fatigue. Therefore, the focus of treatment must not be limited to medication and disease activity but also include promoting a healthier lifestyle, acknowledging the burden of IBD and coping with a chronic disease.

Fatigue, I think, is the common complaint of all people with a chronic disease or autoimmune disorder. That's why it's good to take a more holistic approach, rather than just looking at what medications we're going to give next. [Sarah]

Drawing from their experiences of limited attention for fatigue during medical consultations, many patients expressed a desire for *healthcare professionals to discuss fatigue and offer care actively*. Specifically, patients preferred that healthcare professionals ask about their experience of fatigue and explore together which personal factors might affect fatigue (e.g., diet, physical activity, underlying medical condition or psychological well‐being), in order to determine possible areas for care. Especially patients who did not have the energy to find suitable care themselves said it would be helpful if a healthcare professional would initiate the conversation.

As long as you're not feeling well and you're in the middle of that, you don't have the energy to ask for care. You almost want to be taken by the hand. [Melissa]

Patients expressed several benefits of a healthcare professional initiating the conversation about fatigue. First, it would acknowledge fatigue as a common symptom in IBD and recognise it as a challenging issue to deal with. Second, patients reported that by creating a safe environment to discuss various symptoms, they would feel less vulnerable and ashamed about addressing these concerns. Finally, patients outlined that active discussion by a healthcare professional can assist those who are unaware of treatment options or hesitant to a certain type of treatment in understanding the potential benefits. Altogether, patients explained that this approach may result in earlier access to effective treatment.

#### 5.3.2 Theme 2: What Care to Offer

While some patients expressed uncertainty about what could alleviate their fatigue, others shared preferences for the type of care they would like to receive. Most patients reported a need to receive more *information about fatigue management*, citing their lack of awareness regarding potential treatment options for fatigue. Moreover, they expressed a need to understand possible causes of fatigue beyond inflammation. Since information on the internet was often perceived as too general, patients would prefer to receive tailored information from healthcare professionals encompassing strategies to manage fatigue in IBD, along with guidance on accessing support services. One patient proposed the creation of an online platform featuring tips, information, and a forum for patients to share experiences with other patients, as well as to receive support from a professional.

MyIBDCoach would be a great platform. Especially if there is someone who specializes in fatigue symptoms, who can then guide you or give tips and tricks. [Sandra]

In addition to information provision, many patients emphasised the importance of *eliminating any somatic cause of fatigue* before seeking alternative types of support, such as psychological treatment in coping with fatigue. Moreover, patients expressed a desire to *discuss medication options* with their healthcare professional. Some sought a ‘quick fix’ by adjusting their medication, citing positive experiences with fatigue relief from medications like prednisone or vitamin B12 injections. Conversely, others aimed to minimise medication intake alongside their IBD medication or even discontinue their IBD medication entirely. They believed that their fatigue could be caused by the medication, although they were aware of the increased risk of a flare when medication was discontinued. Apart from IBD medication, several patients showed hesitancy towards sleep medication and antidepressants due to concerns about potential dependency and social stigma:

I'm not someone who gets on medication to sleep, no… I don't want to become dependent on a sleeping pill. [Karen]

Many patients recognised the importance of a healthy lifestyle in managing fatigue and viewed *lifestyle support* as a positive motivator for sustaining healthier habits. They believed that IBD could result in a sensitivity to certain food products, which they associated with abdominal pain and decreased energy levels, and expressed a need for guidance from a dietitian. Moreover, patients suggested that improving physical activity and losing weight, preferably under the supervision of a physiotherapist, could potentially enhance their energy level.

It is important that they have a real look at what we can do about nutrition or physical fitness. Maybe that fatigue won't go away, but we can improve it. [Natalie]

Other patients expressed a wish to address their sleep quality or mentioned that relaxation exercises or massage conducted by a physiotherapist could aid them in coping more effectively with sleep problems and fatigue.

The need for *psychological support* primarily revolved around learning to accept the disease and managing accompanying emotions. Patients believed this could also alleviate the burden of fatigue. It was also mentioned that consultations with a psychologist or coach could assist them in handling daily life with fatigue, including aspects like planning, dealing with challenges and increasing self‐compassion.

I know from myself, and I also hear it from my husband, that I am quite hard on myself. […] I push my fatigue aside, and sometimes he can see that it's just not possible. But then I go on anyway, for the sake of others. That's where a little guidance for me wouldn't hurt. [Sandra]

Some patients had already experienced reductions in fatigue after sessions with a psychologist or through participating in a mindfulness course, reinforcing their belief that psychological support should be part of IBD treatment. Conversely, others stated explicitly that they did not have a need for psychological care. Reasons included negative experiences with psychologists, a sense of self‐sufficiency in managing the disease, the perception that fatigue is a physical rather than a mental issue, or the belief that talking about fatigue will not solve the problem.

Another type of support addressed was *peer support*, which highlights its value in allowing patients to share experiences regarding the impact of fatigue on their lives and how they cope with it.

I would be particularly interested in how others my age combine wanting a career on the one hand and also having a desire to have children on the other with their illness. [Sarah]

While peer support could be facilitated in different ways, several patients proposed the establishment of a peer support group offered by the hospital, possibly supervised by a coach or healthcare professional who has experience with IBD‐related fatigue. A few patients saw little benefit in joining such a group, mostly because they did not like to hear other people's matters.

Finally, two patients primarily experienced a need for *practical support* in times of serious fatigue. They highlighted that assistance with tasks such as garden maintenance, household chores, and childcare could help them recharge if needed. It is noteworthy that both these patients were not living with a partner.

## 6 Discussion

This qualitative study in fatigued patients with IBD aimed to provide insight into their reasons for (not) seeking care for fatigue and their need for care. Patients reported various facilitators and barriers for seeking care. Some of these were related to their own perceptions of (coping with) fatigue, the efficacy of fatigue interventions, knowledge and prior care experiences. Others were related to the knowledge and behaviour of healthcare professionals. In addition, patients' physical and emotional well‐being, social relationships and practical factors played a role in *why* they did (not) seek care for fatigue. When patients were asked about their needs for care to manage fatigue, they reported needs regarding *how* care should be provided and *what* type of care they would find acceptable and beneficial. Altogether, our results indicated that IBD‐related fatigue is a multidimensional issue that, according to patients, requires a holistic and person‐centred treatment approach. The findings underline the importance of physicians and nurses proactively addressing fatigue, thereby facilitating an open dialogue with patients on this subject. Even though some patients did not perceive a need for professional care for fatigue because they were able to manage it themselves, they generally expressed hope for something that could help them manage their fatigue.

Our study addresses the gap in the current literature regarding why patients with IBD are not satisfied with fatigue‐related care. Particularly, their perceptions about fatigue and ways to cope with fatigue were found to play a crucial role in whether to seek and accept care or not. Results highlight individual differences among patients in their perceptions of fatigue and their need for care. For instance, some patients had a need for care because they perceived fatigue as too hindering, whereas others reported a low need for care because they perceived that nothing can be done about fatigue as it is an inherent part of IBD. These individual differences can be understood using Leventhal's common‐sense model of self‐regulation, which proposes that care needs are influenced by patients' subjective perceptions about the causes and consequences of their illness and their coping (Leventhal et al. 2003). Our findings contribute to the evidence supporting this model as found for other symptoms and in other patient samples (Dibley et al. 2021; Richardson et al. 2017; Luca et al. 2022), hereby suggesting generic processes in managing illness and (delay in) care uptake.

Also, patients' perceptions about the availability and effectiveness of fatigue‐related care were found to play a role in seeking care. This finding supports theoretical notions and empirical evidence showing that trust in one's healthcare professional and positive treatment expectations contribute to better health outcomes, including health‐ and care‐seeking behaviours (Leventhal et al. 2003; Birkhäuer et al. 2017). Our findings add to the literature by showing that patients' treatment perceptions can be affected by how they perceive the knowledge and behaviour of their healthcare professionals. For example, when a physician or nurse fails to discuss fatigue or offer treatment options, patients are likely to believe that there are no effective interventions available, which reduces their tendency to discuss fatigue and ask for care. Results imply that healthcare professionals can play an important role in increasing patients' trust in fatigue‐related care and facilitating care‐seeking behaviour, by actively discussing patients' perceptions and preferences, as well as treatment options (Lai et al. 2019). The model of shared decision making as described by Elwyn and colleagues (2012) may be useful in this respect, as it emphasises the importance of building a supportive relationship in the clinical encounter so that information can be shared and patients are supported to express their preferences and views during the decision‐making process. This is in line with the finding that patients, while acknowledging their own responsibility to raise concerns about fatigue during consultations, express a clear need for more guidance from healthcare professionals to effectively discuss and manage fatigue (Schoefs et al. 2023; Feeney et al. 2022).

Regarding the type of care they would like for managing fatigue, patients emphasised the necessity of a holistic and person‐centred approach that addresses individual needs and preferences. Our results suggest that patients with IBD prefer a twofold approach to managing fatigue. First, eliminating the possibility of a physical cause, and second, providing education about fatigue and discussing the range and efficacy of interventions, including lifestyle‐, psychological‐ and practical support. Patients perceive fatigue as a multidimensional symptom, influenced by factors beyond their medical illness, which may vary among patients, thus requiring a personalised approach. A potentially useful framework for understanding and addressing IBD‐related fatigue is the biopsychosocial approach (Artom et al. 2016), which recognises the interplay of biological, psychological and social factors in fatigue. Utilising a biopsychosocial perspective resonates strongly with the broader notion that IBD care should adopt an integrated multidisciplinary approach (Feeney et al. 2022; Mikocka‐Walus et al. 2014).

### 6.1 Strengths and Limitations of the Work

One of the key strengths of the present study lies in its qualitative design, enabling a rich and thorough understanding of the process of care‐seeking for IBD‐related fatigue. This approach facilitated a comprehensive overview of patients' perspectives and experiences with fatigue‐related care, which would not have been possible with a quantitative study design. Another strength is that we screened patients for fatigue, ensuring that they had relevant experiences with the study topic. The interdisciplinary composition of our research team, comprising both psychologists and physicians, provided a valuable combination of diverse yet complementary perspectives. While the psychologists offered insights grounded in theoretical frameworks on care‐seeking, the physicians contributed largely to explaining the clinical relevance of the results within the context of patient care. This collaborative approach ensured a robust and clinically meaningful interpretation of the data, bridging theory [e.g., Leventhal's (Leventhal et al. 2003) common‐sense model of self‐regulation and the model of shared decision making by Elwyn and colleagues (2012)] with practical implications for clinical practice.

Despite these strengths, it is important to consider several limitations when interpreting the results. A first limitation concerns the study participants, as the study included more females than males despite employing a purposive sampling strategy. As such, the perspective of male patients may be underrepresented in our findings. This discrepancy might be attributed to the fact that, based on the screening for severe fatigue, more females than males were invited for our study. However, given that fatigue is generally more prevalent in females (Schreiner et al. 2021), we can assume that the perspective of fatigued patients with IBD is represented sufficiently. Second, we were unable to examine differences in care‐seeking and needs among patients of different ages, with varying comorbidities or a different time since diagnosis. The literature indicates that younger patients with IBD and those with a recent diagnosis often experience more severe fatigue compared to older patients or those with a longer disease duration (Schreiner et al. 2021). In terms of time since diagnosis, our sample was homogenous, with a minimum duration of six years. As a result, the findings may not directly apply to patients with a more recent diagnosis. However, patients did indicate the importance of tailoring care to the stage of the disease. Regarding age, our study included a wide range of patients, which enhances the generalisability of the results. Nonetheless, this broad range limited our ability to explore differences in the identified themes across age groups. Further quantitative research involving a larger number of patients is needed to statistically examine the role of age, comorbidities and time since diagnosis in patients' care needs and preferences. Third, the data analysis was not performed simultaneously with data collection, meaning that the interview guide was not adjusted for subsequent interviews based on emerging issues from earlier interviews. For example, the role of social relationships and support was only mentioned by a few participants and interestingly only as a facilitator rather than a barrier (e.g., others reinforcing not to seek care and passivity, by taking over tasks and protecting the patient). These aspects might have been more prominent had they been given greater consideration during the interviews. Nonetheless, based on the richness of data and the fact that no new information came up in the last interviews, we believe that the most significant themes were explored sufficiently.

### 6.2 Clinical Implications and Future Research

The current results provide several implications for improving the management of IBD‐related fatigue. To facilitate an open conversation about fatigue, physicians and nurses are requested to proactively address the topic of fatigue and provide adequate information regarding possible causes of fatigue, treatment options, and their efficacy. Especially patients who hold illness‐ or treatment perceptions that may hinder adequate care seeking and uptake may benefit from such information provision. Considering the limited time available during medical consultations, screening for fatigue and the need to discuss it may be performed prior to the consultation, using a short questionnaire or online monitoring tool. Moreover, patients may be referred to an external source (e.g., leaflet or website), of which the content is then discussed during a focused and structured follow‐up session. Future research is needed to examine how information about fatigue and its' management is best provided to patients with IBD. Moreover, since the literature indicates that healthcare professionals often do not know how to manage IBD‐related fatigue (Czuber‐Dochan, Norton, Bredin, et al. 2014), education of physicians and nurses should also receive more attention. In order to optimise this education, future studies could examine healthcare professionals' (mis)perceptions about fatigue.

The current findings emphasise the need for a clearly defined care path for IBD‐related fatigue, describing the responsibilities of both healthcare professionals and patients, and providing information about possible explanations for fatigue and available treatment modalities. Keefer and colleagues (2022) have presented practical recommendations for integrating fatigue and other psychosocial aspects of IBD in the management of IBD. In our study, patients expressed a need for a holistic and patient‐centred care approach, including psychological treatment and lifestyle support. Regarding the effectiveness of psychological and lifestyle interventions, literature shows that distinct types of interventions may be helpful for patients with IBD experiencing fatigue. For example, cognitive‐behavioural therapy and mindfulness‐based interventions (Emerson et al. 2021; Bredero et al. 2023), as well as structured exercise programs (Jones et al. 2022), seem promising and have been shown to be effective in reducing fatigue. Moreover, as already outlined by the second N‐ECCO consensus statement (Kemp et al. 2018), especially nurses could play a central role in this, as they are well‐positioned to adopt a holistic approach to fatigue. Future research is recommended to examine the implementation and effectiveness of incorporating such a holistic care model for IBD‐related fatigue.

## 7 Conclusion

This qualitative study shows that patients with IBD have various perceptions concerning fatigue, ways to manage fatigue, the availability and efficacy of fatigue‐related care, and the knowledge and behaviour of healthcare professionals, which may encourage or withhold them from seeking care. Some of these perceptions can be maladaptive and can effectively be addressed by providing adequate information about fatigue and treatment options. Our finding that the behaviours of healthcare professionals play an important role in the decision to seek care for fatigue underlines the importance of physicians and nurses proactively addressing fatigue to foster open discussions with patients with IBD. Moreover, the varying needs expressed by patients regarding the provision of fatigue‐related care and the type of interventions deemed beneficial support the notion that IBD‐related fatigue is a multidimensional symptom that requires a holistic and person‐centred treatment approach.

## Author Contributions

**Quirine M. Bredero:** conceptualisation, methodology, project administration, investigation, data curation, formal analysis, interpretation of the data, writing original draft, reviewing and editing manuscript. **Milou M. Ter Avest:** formal analysis, interpretation of the data, writing original draft, reviewing and editing manuscript. **Gerard Dijkstra:** conceptualisation, resources, reviewing and editing manuscript, supervision. **Joke Fleer:** conceptualisation, methodology, interpretation of the data, reviewing and editing manuscript, supervision. **Maya J. Schroevers:** conceptualisation, methodology, interpretation of the data, reviewing and editing manuscript, supervision. All authors had full access to the data in the study. All authors have agreed on the final version and meet at least one of the following criteria (recommended by the ICMJE): (1) substantial contributions to conception and design, acquisition of data, or analysis and interpretation of data; (2) drafting the article or revising it critically for important intellectual content.

## Ethics Statement

The study was approved by the Institutional Review Board of the University Medical Center Groningen (UMCG) on July 12, 2021, registered as no. 2021/329, and the study has been performed in accordance with the principles of the Declaration of Helsinki (2013). Any data utilised in the submitted manuscript have been lawfully acquired in accordance with The Nagoya Protocol on Access to Genetic Resources and the Fair and Equitable Sharing of Benefits Arising from Their Utilization to the Convention on Biological Diversity.

## Consent

All patients provided written informed consent for their participation in the study.

## Conflicts of Interest

G.D. received a research grant from Royal DSM and advisory board fee from Pharmacosmos outside the submitted work. The other authors declare that they have no conflicts of interest.

## Peer Review

The peer review history for this article is available at https://www.webofscience.com/api/gateway/wos/peer‐review/10.1111/jan.16837.

## Supporting information


**Supporting Information 1.** COREQ checklist.

**Supporting Information 2.** Final template.

**Supporting Information 3.** Illustrative quotes for each (sub)theme.

## Data Availability

The data that support the research findings are available upon reasonable request to the corresponding author. Data are not publicly available due to privacy and ethical restrictions.
