# Supplementary material for: Reasons for (Not) Seeking Care for Fatigue and Care Needs Among Patients With Inflammatory Bowel Disease: A Qualitative Interview Study
Source: J Adv Nurs. 2025 Feb 25;81(10):6815–28. doi: 10.1111/jan.16837 (PMC12460950; doi:10.1111/jan.16837)
Supplement: Supplementary file 2 — Supporting Information 2. Final template. [file JAN-81-6815-s001.docx]

**Supplementary File 2 - Final template**

**Reasons for (not) seeking care: Facilitators and barriers**

1. **Cognitions about fatigue and coping**
   - **Facilitators**
     - *Fatigue*
       - Perceiving fatigue as too hindering
     - *Coping*
       - Desiring to improve functioning
       - Perceiving to be unable to deal with fatigue independently
       - Seeking a medical explanation for fatigue
   - **Barriers**
     - *Coping*
       - Perceiving to have learned to live with fatigue
         - Acceptance of fatigue
         - Fatigue is not hindering enough
         - I know how to deal with fatigue
       - Desiring to deal with fatigue independently
       - Denying having a chronic disease
         - Avoiding confrontation with illness
         - Not listening to your body
2. **Perceptions of fatigue-related care and previous care experiences**
   - **Facilitators**
     - Having trust in the effectiveness of available care
   - **Barriers**
     - Having limited trust in the effectiveness of available care
       - Having negative care experiences
       - Doubting whether it is worth the investment
     - Perceiving care needs are fulfilled
     - Perceiving that nothing can be done about fatigue
     - Having a lack of knowledge
       - Not thinking about care for fatigue
       - Not knowing how to seek care
       - Not knowing what can help
3. **Perceived knowledge and behaviour of healthcare professionals**
   - **Facilitators**
     - Perceiving fatigue as a topic of conversation
   - **Barriers**
     - Perceiving that fatigue is not taken seriously by healthcare professionals
     - Perceiving that fatigue-related care is not (adequately) offered
     - Perceiving lack of knowledge in healthcare professionals regarding how to deal with fatigue
4. **Physical and emotional well-being**
   - **Facilitators**
     - Experiencing (other) physical symptoms
     - Experiencing an unhealthy lifestyle
   - **Barrier**
     - Experiencing IBD-related physical symptoms
     - Feeling emotionally unfit
5. **Social relationships and support**
   - **Facilitators**
     - Perceiving negative impact of fatigue on relationships
     - Receiving advise from others to seek care
6. **Practical factors**
   - **Barriers**
     - Perceiving practical barriers

**Care needs for IBD-related fatigue**

1. **How to offer care**
   - Taking a person-centred approach
     - Type of care
     - Timing of care
     - Location of care
   - Taking a holistic care approach
   - Healthcare professionals should discuss fatigue and offer care actively
     - More attention for fatigue
     - Active offer of care
     - Accessible and safe contact with healthcare professional
2. **What care to offer**
   - Information provision on fatigue management
   - Eliminating physical causes for fatigue
   - Discussing medication options
     - As little medication as possible
     - Changing medication
   - Lifestyle support
     - Dietary support
     - Support for improving sleep
     - Physical activity support
     - Relaxation
   - Psychological support
     - Psychological care or coaching
     - No psychological care
   - Peer support
   - Practical support
