# Supplementary material for: Reasons for (Not) Seeking Care for Fatigue and Care Needs Among Patients With Inflammatory Bowel Disease: A Qualitative Interview Study
Source: J Adv Nurs. 2025 Feb 25;81(10):6815–28. doi: 10.1111/jan.16837 (PMC12460950; doi:10.1111/jan.16837)
Supplement: Supplementary file 3 — Supporting Information 3. Illustrative quotes for each (sub)theme. [file JAN-81-6815-s003.docx]

**Supplementary File 3 – Illustrative quotes for each (sub)theme**

**Reasons for (not) seeking care: Facilitators and barriers**

| **Theme** | **Category** | **Barrier/facilitator** | **Subcode** | **Illustrative quote** |
| --- | --- | --- | --- | --- |
| **1. Cognitions about fatigue and coping** | **Facilitators**  *Fatigue* | Perceiving fatigue as too hindering |  | *“If I really just can’t get out of bed because I’m just too tired, and that the flare-up is just extremely bad again, that I’m really feeling down because of that ... Yes, then I would say: ‘What can we do about it?’”* [Laura] |
|  | **Facilitators**  *Coping* | Desiring to improve functioning |  | *“I want to have it as optimal as possible, so I always try to take that step to get back towards normal. Not so much in terms of illness, but just in terms of functioning, in daily activities and things you want to do. ... You don’t want the disease to take over in that.”* [Thomas] |
|  |  | Perceiving to be unable to deal with fatigue independently |  | *“Especially the consideration that I can’t do that alone. I was thinking I couldn't see the forest for the trees. What do I need to do to feel better?’”* [Linda] |
|  |  | Seeking a medical explanation for fatigue |  | *“I also had a test done via the general practicioner, just for sleep apnea, because they thought maybe the problem is quality of sleep. Nothing came out of it.”* [Daniel] |
|  | **Barriers**  *Coping* | Perceiving to have learned to live with fatigue | Acceptance of fatigue | *“You know, the fatigue is just there and I’m not fighting it. It’s there. [...] And at some point you do come to a kind of resignation, I guess.”* [Natalie] |
|  |  |  | Fatigue is not hindering enough | *“It’s always bothering me, it’s not something I’ve had trouble with just recently. So I’ve kind of learned to live with it actually. In that sense, it doesn't hinder me so much that I would go and get help for that.”* [Rachel] |
|  |  |  | I know how to deal with fatigue | *“You try to stay in a rhythm yourself, with food and things like that. Yes, that does work, of course. You can feel for yourself what works and what doesn't.”* [Sandra] |
|  |  | Desiring to deal with fatigue independently |  | *“I don’t want to ask for it, I guess. I feel like a bit of a nag when I ask if someone can help me with my fatigue. I think someone’s reaction would be: ‘Yes, but you can do something about that yourself, can’t you? You can watch your diet and exercise, can’t you? Why, what kind of help are you looking for?’ If someone offers it, yes of course, but I would never ask for it.”* [Rachel] |
|  |  | Denying having a chronic disease | Avoiding confrontation with illness | *“I’d rather immerse myself in the things I enjoy, in hobbies and so on, than another medical problem, or another thing I don’t really want to deal with.”* [Natalie] |
|  |  |  | Not listening to your body | *“Perhaps the barrier is that you don’t want to admit you are sick. You just carry on, when in fact, it would be better not to.”* [Melissa] |
| **2. Perceptions of fatigue-related care and previous care experiences** | **Facilitators** | Having trust in the effectiveness of available care |  | *“If it’s really something with a high chance of success, it would be different. But to go somewhere and they don’t really know what to do with you, that’s a little tricky.”* [Brian] |
|  | **Barriers** | Having limited trust in the effectiveness of available care | Having negative care experiences | *“Everything goes wrong with me very often, so that doesn’t exactly give me a sense of confidence either.”* [Mary] |
|  |  |  | Doubting whether it is worth the investment | *“It should not take too much energy to undergo it. Because if it gives you only 70%, then on balance, you don’t gain anything.”* [Hannah] |
|  |  | Perceiving care needs are fulfilled |  | *“I don’t benefit from that at the moment, nor do I think in the future, because I feel I got the help I was looking for.”* [Daniel] |
|  |  | Perceiving that nothing can be done about fatigue |  | *“They have been saying all the time: ‘This fatigue, it’s just part of it [IBD] and we don’t really know what to do with it.’ So you just push that aside first, I guess.”* [Natalie] |
|  |  | Having a lack of knowledge | Not thinking about care for fatigue | *“I've always taken care of myself, so it doesn’t occur to you then to ask for help for certain things.”* [Mary] |
|  |  |  | Not knowing how to seek care | *“I never thought about it, because I wouldn’t know who to turn to. There’s not much information about it, in my opinion. I haven't seen or found that yet”* [Sandra] |
|  |  |  | Not knowing what can help | *“It [the fatigue] is also something so elusive. So no, I wouldn't know what to look for.”* [Hannah] |
| **3. Perceived knowledge and behaviour of healthcare professionals** | **Facilitators** | Perceiving fatigue as a topic of conversation |  | *“From the moment I ended up in the [name of hospital], my request for help for fatigue came up a lot more. I was actually advised in that by the doctor himself. Those questions were asked much more concretely here, compared to in my previous hospital.”* [Thomas] |
|  | **Barriers** | Perceiving that fatigue is not taken seriously by healthcare professionals |  | *“Of course, during check-ups they always ask how you feel. I always indicate that I’m tired, but they either don't respond or they say ‘Well, that's part of it.’ [...] So I didn't really ask for it, no.”* [Rachel] |
|  |  | Perceiving that fatigue-related care is not (adequately) offered |  | *“There is also very little about it and very little offered for it. I mean, at the hospital they don’t really offer anything for fatigue.”* [Sandra] |
|  |  | Perceiving lack of knowledge in healthcare professionals regarding how to deal with fatigue |  | *“Often I hear from them: ‘Yes okay, it’s too bad you have that, but we’re not so sure what to do with that either...’”* [Nicole] |
| **4. Physical and emotional well-being** | **Facilitators** | Experiencing (other) physical symptoms |  | *“Well, I had such terrible physical pains. And I’ve had that for a long time. I already started to walk more crookedly. I just couldn’t do my job anymore, and so at a certain point you just get stuck physically, but also mentally.”* [Jennifer] |
|  |  | Experiencing an unhealthy lifestyle |  | *“In the time I’ve been at home, I’ve gained a little bit of weight. So I also did seek help from dietitians, as I’m trying to lose some weight. Hopefully that also helps with getting a little more energy.”* [Daniel] |
|  | **Barriers** | Experiencing IBD-related physical symptoms |  | *“Because of my Crohn’s disease, I also have to schedule it [ receiving care]. For example, tomorrow I have to be at my physical therapist at 9 a.m., then I get up at 5:30 a.m. Otherwise that won’t work out. I try to schedule everything in the afternoon or at the end of the morning. And that doesn’t lower the threshold to seek care either.”* [Mary] |
|  |  | Feeling emotionally unfit |  | *“So when things are back on track a little, there will be room for me to undertake things again. But if I’m not feeling well, physically or mentally, there’s no room to take the initiative.”* [Melissa] |
| **5. Social relationships and support** | **Facilitators** | Perceiving negative impact of fatigue on relationships |  | *“I noticed that it was all taking too long and that I was burdening my family. As soon as I notice it's getting really annoying, also for everyone around me, I'm going to make an effort to get help.”* [Melissa] |
|  |  | Receiving advise from others to seek care |  | *“On other people’s advice actually. My mother knew someone who had Crohn's disease. She was also in therapy with that person and they had achieved very good results.”* [Thomas] |
|  | **Barriers** | n/a |  | n/a |
| **6. Practical factors** | **Facilitators** | n/a |  | n/a |
|  | **Barriers** | Perceiving practical barriers |  | *“That [care for fatigue] is mostly in the south of the country. There’s actually too little here in the north. I am not going to [city in the south] just because we’re all going for a walk there. I would love to, but no, that’s too far for me.”* [Nicole] |

**Care needs for IBD-related fatigue**

| **Theme** | **Subtheme** | **Subcode** | **Illustrative quote** |
| --- | --- | --- | --- |
| **1. How to offer care** | Taking a person-centred approach | Type of care | *“On the internet there is plenty to find. You are overloaded with all kinds of success stories and that just makes you sad. You don’t know how to handle it. Where to start? ... You just want what’s right for you.”* [Natalie] |
|  |  | Timing of care | *“So if the question whether there are things that come into play, is actually already being asked of you, particularly in the beginning of this disease, at the time of your diagnosis, that’s a pretty good one.”* [Thomas] |
|  |  | Location of care | *“Anything that can be done at home, is close by, or doesn’t take too much time, would help. But no, I’ve never found that.”* [Hannah] |
|  | Taking a holistic care approach |  | *“Fatigue, I think, is the common complaint of all people with a chronic disease or autoimmune disorder. That’s why it’s good to take a more holistic approach, rather than just looking at what medications we’re going to give next.”* [Sarah] |
|  | Healthcare professionals should discuss fatigue and offer care actively | More attention for fatigue | *“When you’re in consultation with a doctor, that they actually look at what we can do nutritionally or to make the conditions as good as possible. Maybe that fatigue won’t go away, but we can improve it. It’s kind of an under-addressed problem.”* [Natalie] |
|  |  | Active offer of care | *“As long as you’re not feeling well and you’re in the middle of that, you don’t have the energy to ask for care. You almost want to be taken by the hand.”* [Melissa] |
|  |  | Accessible and safe contact with healthcare professional | *“Also a doctor who facilitates that, who is open in that himself and dares to ask the question whether there might be more things at play. It is a confidential counsellor after all. It is a confidential conversation and what you discuss with a doctor can basically be anything. Anything that affects you on your illness.”* [Thomas] |
| **2. What care to offer** | Information provision on fatigue management |  | *“MyIBDCoach would be a great platform. Especially if there is someone who specializes in fatigue symptoms, who can then guide you or give tips and tricks.”* [Sandra] |
|  | Eliminating physical causes for fatigue |  | *“Yes and before I want to take those next steps of finding support in dealing with fatigue, I actually want answers to those medical questions.”* [Thomas] |
|  | Discussing medication options | As little medication as possible | *“I’m not someone who gets on medication to sleep, no... I don’t want to become dependent on a sleeping pill.”* [Karen] |
|  |  | Changing medication | *“Well, suppose we can adjust the medications in such a way that they work well, that I am less tired, or that I suffer less. Suppose he says, ‘Yeah, that 10 mg of prednisone will do, too.’ But by now I know that the prednisone wears me out. He often asks when the medication can go down, so I don’t expect him to do that.”*[Laura] |
|  | Lifestyle support | Dietary support | *“I'm interested in the effect of nutrition on all of this, because obviously it has to do with your gut. I would like to know if there is anything that can be done in that.”* [Sarah] |
|  |  | Support for improving sleep | *“If they have exercises I can try before bed so that I fall asleep, I would really like to try them.”* [Karen] |
|  |  | Physical activity support | *“It is important that they have a real look at what we can do about nutrition or to improve fitness. Maybe that fatigue won’t go away, but we can improve it.”* [Natalie] |
|  |  | Relaxation | *“What I do every so often is I go to a Shiatsu massage, because I actually have to consciously seek relaxation, otherwise I won't get that tension out of my body.”* [Sarah] |
|  | Psychological support | Psychological care or coaching | *“I know from myself, and I also hear it from my husband, that I am quite hard on myself. [...] I push my fatigue aside, and sometimes he can see that it’s just not possible. But then I go on anyway, for the sake of others. That’s where a little guidance for me wouldn’t hurt.”* [Sandra] |
|  |  | No psychological care | *“Because I feel like the problem is not mental, so I don’t think there is a lot of room for improvement there.”* [Daniel] |
|  | Peer support |  | *“I would be particularly interested in how others my age combine wanting a career on the one hand and also having a desire to have children on the other with their illness.”* [Sarah] |
|  | Practical support |  | *“The fatigue and the Crohn’s are always with you, so the only thing that helps me to start again, to recharge again, is more practical help anyway. Someone else who actually does things for you, care for your child, housework or just that you’re kind of forced to relax yourself.”* [Hannah] |
